# Outstanding Reviewers for *RSC Advances* in 2023

**DOI:** 10.1039/d4ra90064b

**Published:** 2024-07-01

## Abstract

We would like to take this opportunity to thank all of *RSC Advances*' reviewers for helping to preserve quality and integrity in chemical science literature. We would also like to highlight the Outstanding Reviewers for *RSC Advances* in 2023.

## Abstract 2

We would like to take this opportunity to highlight the Outstanding Reviewers for *RSC Advances* in 2023, as selected by the editorial team for their significant contribution to the journal.

## Graphical abstract

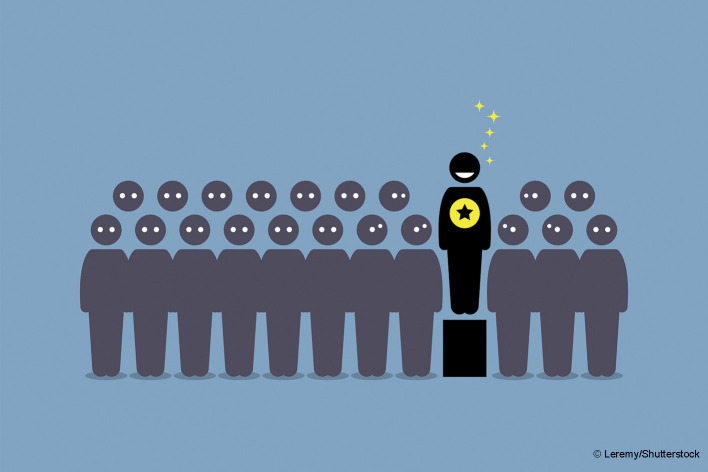

Each one of our outstanding peer reviewers has been carefully selected by our editorial team and the list includes active researchers who have made significant contributions to peer review and have gone above and beyond in their actions.

In recognition of the varied contributions of our reviewer community, our Outstanding Reviewers from 2023 have been chosen based on several different measures, including the number, timeliness and quality of the reports completed over the last 12 months. We have also chosen to highlight reviewers who provided exceptionally thorough and detailed reports and reviewers who made additional efforts to aid authors in improving their manuscripts.

We would also like to direct a special thanks to the members of the *RSC Advances* Reviewer Panel for their hard work and dedication, and the valuable contribution they have made to the journal. The Reviewer Panel is a key part of our commitment to deliver rigorous and fair peer review and ensures that manuscripts are handled by experts throughout the peer review process. We are proud to work with these individuals and recognise their crucial role for the journal.

*RSC Advances* strives to represent the diversity of the chemistry community, and we would like our reviewer community to reflect that. We are always looking to improve the diversity of our reviewer community in all its forms, and welcome applications for new reviewers through the RSC's Become a Reviewer page.

In 2023 we introduced Transparent Peer Review (TPR) to *RSC Advances*. We firmly believe this will support trust and rigour in our peer review processes, as well as improving the quality of reviewer reports for our authors. We are already seeing a great level of engagement, with over a third of our authors choosing to publish the reviewer comments alongside their paper. For more information on TPR, read our editorial: https://pubs.rsc.org/en/content/articlelanding/2023/ra/d3ra90060f

“*Peer review is a vital aspect of ensuring the quality and originality of the research published in RSC Advances. Over the last year we have introduced transparent peer review to improve the transparency of the peer review process and support ways to improve the quality of peer review. We thank all the reviewers that continue to support RSC Advances and very much appreciate their contributions, especially in the face of ever-increasing workloads.*” – Karen Faulds, Editor-in-Chief

“*Our outstanding reviewers reflect the RSC Advances community: international, diverse, committed and outstanding experts in their scientific areas. I very much thank them for their dedication to the review process over the past year and their contributions to the continued success of the journal.*” – Russell J. Cox, Editor-in-Chief

 

***RSC Advances* 2023 Outstanding Reviewers:**

 

Professor Gerard Alonso

University of Concepción

ORCID: 0000-0001-8162-9270

 

Dr Aziz-Ur-Rahim Bacha

Fudan University

ORCID: 0000-0003-2060-6176

 

Dr Suvendu Biswas

Northwestern University

ORCID: 0000-0002-1348-2358

 

Dr Filippo Campana

University of Perugia

 

Dr Scott Crawford

National Energy Technology Laboratory

ORCID: 0000-0003-2161-6536

 

Dr Chaoying Ding

University of Delaware

ORCID: 0000-0002-6722-4821

 

Dr Shipeng Ding

Southeast University

ORCID: 0000-0002-0065-1554

 

Dr Flavio Figueira

University of Aveiro

ORCID: 0000-0002-3685-9736

 

Dr Fateh Gill

Graphic Era Deemed to be University

 

Dr Branko Kordić

University of Novi Sad

ORCID: 0000-0003-2366-1277

 

Dr Xiuling Liu

Michigan Technological University

ORCID: 0000-0002-6685-8531

 

Dr Priscilla Mendonca Matos

University of Nottingham

ORCID: 0000-0002-2982-5909

 

Dr Mirko Maturi

University of Cadiz

ORCID: 0000-0003-3176-0697

 

Dr Christian Nkanga

Université de Kinshasa

ORCID: 0000-0001-7712-478X

 

Dr Chunxiang Wang

Takeda Pharmaceutical Co Ltd

ORCID: 0000-0002-8669-1767

 

Dr Luning Wang

Stanford University

ORCID: 0000-0002-3908-7946

 

Dr Hao Yan

China University of Petroleum East China

ORCID: 0000-0003-1531-3053

 

Dr Yijie Yin

University of California San Diego

ORCID: 0000-0002-6472-9293

 

Dr Kaisong Yuan

Shantou University Medical College

ORCID: 0000-0003-3978-0908

 

Professor Bin Zhang

Shenzhen University First Affiliated Hospital

ORCID: 0000-0002-2112-2555

 

Professor Yu Zhou

Nanjing Tech University

ORCID: 0000-0003-1757-3705

 

Dr Yifan Zhu

Rice University

ORCID: 0000-0002-9816-5764

 

***RSC Advances* Reviewer Panel 2023 Outstanding Reviewers**

 

Dr Sohini Bhattacharyya

Rice University

ORCID: 0000-0002-4626-1578

 

Dr Angel Bracamonte

National University of Cordoba

ORCID: 0000-0003-4760-3872

 

Dr Wanhao Cai

University of Freiburg

ORCID: 0000-0002-3466-8530

 

Dr Shishir Chourey

Bristol-Myers Squibb Co R&D Lawrenceville

ORCID: 0000-0002-2629-9632

 

Assistant Professor Sherif Fahmy

University of Hertfordshire-Egypt Campus

ORCID: 0000-0003-3056-8281

 

Dr Hanuman Kalmode

Scientist-II, Abzena LLC

ORCID: 0000-0002-1577-5103

 

Dr Artur Kasprzak

Politechnika Warszawska Wydzial Chemiczny

ORCID: 0000-0002-4895-1038

 

Dr S. Girish Kumar

RV College of Engineering

ORCID: 0000-0001-9132-1202

 

Dr Shota Kuwahara

Toho University

ORCID: 0000-0001-6095-0866

 

Dr Feng Li

University of Sydney

ORCID: 0000-0003-4448-074X

 

Dr Hu Li

Guizhou University

ORCID: 0000-0003-3604-9271

 

Professor Jian-Min Li

Zhejiang University

ORCID: 0000-0002-3917-8653

 

Dr Yixuan Li

Tesla Motors Inc

ORCID: 0000-0003-1589-0395

 

Professor Ekkehard Lindner

University of Tubingen

 

Dr Jakub Matusiak

Lublin University of Technology

ORCID: 0000-0002-0797-1737

 

Professor Maria Vesna Nikolic

University of Belgrade – Institute for Multidisciplinary Research

ORCID: 0000-0001-5035-0170

 

Dr Wojciech Pajerski

InnoRenew CoE

ORCID: 0000-0002-2336-5550

 

Dr Abhispa Sahu

SiOxMed, LLC

ORCID: 0000-0002-3223-7577

 

Dr Anindita Sarkar

University of Michigan

ORCID: 0000-0002-7947-860X

 

Professor James Sheehan

The University of Alabama

ORCID: 0000-0001-5548-8099

 

Dr Ajay B. Urgunde

Auburn University

ORCID: 0000-0001-8377-5693

 

Professor Rafael Vargas-Bernal

University of Guanajuato

ORCID: 0000-0003-4865-4575

 

Dr Lushun Wang

Stanford University

ORCID: 0000-0002-8373-2199

 

Professor Masanori Yamamoto

Tokyo Institute of Technology

ORCID: 0000-0001-8473-7015

 

We would also like to thank the *RSC Advances* Editorial Board and the chemistry community for their continued support of the journal, as authors, reviewers and readers.

We continue to work on improving the diversity of our reviewer pool to reflect the diversity of the communities that we serve.

 

Laura Fisher, Executive Editor

Karen Faulds, Editor-in-Chief

Russell J. Cox, Editor-in-Chief

